# Treatment of multiple liver metastasis from gastric carcinoma

**DOI:** 10.1186/1477-7819-5-70

**Published:** 2007-06-21

**Authors:** Hitoshi Ojima, Sayaka Ootake, Takehiko Yokobori, Yasushi Mochida, Yasuo Hosouchi, Yasuji Nishida, Hiroyuki Kuwano

**Affiliations:** 1 Department of Surgery, Gunma Prefecture Saiseikai-Maebashi Hospital, 564-1 Kami-shinden, Maebashi, Gunma 371-0821, Japan; 2 Department of General Surgical Science, Gunma University Faculty of Medicine, 3-39-22 Showa-machi, Maebashi, Gunma 371-8511, Japan

## Abstract

**Background:**

The efficacy of operative resection of liver metastasis from colorectal cancer has been established. However, a treatment for liver metastasis from gastric cancer has not yet been established. In this study, we evaluated the efficacy of hepatic arterial infusion for synchronous hepatic metastasis from gastric cancer.

**Patients and methods:**

This study consisted of 37 patients [HAI group; 18 and non-HAI group; 19] with synchronous multiple liver metastases from gastric cancer at Gunma Prefecture Saiseikai-Maebashi Hospital. We retrospectively analyzed the efficacy of HAI.

**Results:**

Response rate (CR + PR) of HAI was 83%. However, HAI treatment did not affect any improvement in the survival rate.

**Conclusion:**

HAI is an effective treatment for control of liver metastasis specifically. The factor effective for an improvement in the survival rate was possibly that of gastrectomy.

## Background

The presence of liver metastasis is one of the most important prognostic factors in patients with gastrointestinal cancer. Gastric cancer with liver metastasis is a non-curable, fatal disease with a 5-year survival of less than 10% [1-4]. The efficacy of operative resection of liver metastasis from colorectal cancer has been established [5-7]. However, the effectiveness of liver resection for metastasis from gastric cancer remains unknown [8-10]. Generally, these patients should receive systemic chemotherapy apart from surgical resection. Some new active anticancer drugs and effective combination regimens have been reported [11-14]. As one of the critical factors determining prognosis is liver metastasis, local control is considered most important.

Recently, advances in vascular interventional radiology have made it easier to insert a catheter percutaneously. Connecting a catheter indwelling in the hepatic artery with a subcutaneously implanted port system facilitates repeated hepatic arterial infusion (HAI) on an outpatient basis [15]. Hepatic metastasis of gastric cancer might possibly be controlled as easily as that of colorectal cancer. However, the development of extrahepatic lesions during HAI therapy could be an important factor determining prognosis [16]. We evaluated the efficacy of hepatic arterial infusion for synchronous hepatic metastasis from gastric cancer.

## Methods

This study consisted of 37 patients (30 (81%) males and 7 (19 %) females; mean age 70.5 ± 8.5 years; range, 54 to 85 years) who had all undergone treatment for synchronous multiple liver metastases from gastric cancer between January 1, 1996 and December 31, 2004 at Gunma Prefecture Saiseikai-Maebashi Hospital.

HAI was performed in our hospital from April, 2001. Our treatment policy for gastric cancer with multiple liver metastases is first to operate on the primary lesion and then HAI is instituted. We perform gastrectomy to maintain QOL if the performance status is good. However, neither an operation nor HAI can sometimes be undertaken. The reasons for which operations were not performed for the study patients were as follows; aged over 85 years (4 patients), and peritonitis carcinomatosa with ascites, Virchow metastasis, multiple lung metastasis, and did not agree to an operation (one each). 18 patients underwent hepatic arterial infusion (HAI) of 5-fluorouracil (5-FU). The remaining 19 patients underwent other treatments, excepting HAI, or were not treated. The reasons for which we did not perform HAI were as follows; 15 patients were cases seen before April, 2001, while in 4 patients the insertion of a catheter was impossible for various reasons. Fifteen patients underwent systemic chemotherapy with tegafur (2 cases), 5-Fluorouracil/cisplatin (5-FU/CDDP) (10 patients) or S-1 (3 patients). Four of the remaining patients were not treated because they were 85 years or older. HAI was performed by repeated HAI chemotherapy employing an implantable port system. The right gastric artery and posterior superior pancreatico duodenal artery were embolized with a metal coil and a catheter inserted from the right femoral artery with its tip located in the gastroduodenal artery. We made a side entrance on the catheter that was opened from the tip for about 3 cm. The side entrance was located in the proper hepatic artery. When gastrectomy was performed, embolization of the right gastric artery was omitted. An arterial implantable port was inserted subcutaneously in the thigh. The regimen was as follows: 5-FU (once 500 mg/body week for 2 hours). CT of the liver was obtained every 3 months, and efficacy was evaluated as complete response (CR), partial response (PR), no change (NC) and progressive disease (PD). HAI was continued if the evaluation after three months showed it to be effective. HAI was essentially undertaken on an outpatient basis. All patients had synchronous and multiple liver metastases from gastric cancer; we excluded metachronous and/or single liver metastasis from gastric cancer. Therefore, there were no adjustments for resection of the liver. One patient survived for more than 5 years without any signs of recurrence.

Survival was analyzed by the Kaplan-Meier method. Multivariate analysis was performed by the log-rank test and statistical significance was determined by *P *< 0.05.

## Results

Table 1 presents the patient characteristics. There were differences in the depth of invasion between the two groups. All in the HAI group and 11 (58%) in the non-HAI group had undergone gastrectomy. The diameters of liver metastases in the HAI group were significantly smaller than those in the non-HAI. The median survival time (MST) was 19.2 months in the HAI group and 8.8 months in the non-HAI. There were no significant differences between the two groups.

**Table 1:** Characteristics of patients

	HAI	non-HAI	*p*-value
n	18	19	
Age	65.9 ± 6.1	73.6 ± 10.2	0.1882
Gender M/F	17/1	13/6	0.0518
Depth of invasion			
T1	1	0	
T2	10	2	0.0111
T3	3	9	
T4	4	2	
Unknown	0	6	
Lymphnode metastasis			
N0	3	0	
N1	8	4	0.2486
N2	4	6	
N3	3	1	
Unknown	0	8	
Location			
U	5	1	
M	10	14	0.1422
L	3	4	
Operation			
+	18	11	0.0024
-	0	8	
Metastasis			
lung	2	1	
Virchow	1	1	0.8281
peritoneum	2	1	
other	1	0	
Diameter of Liver metastasis (cm)	5.8 ± 1.9	7.7 ± 3.7	0.04137
Median survival (mon)	19.2	8.8	0.09596

Table 2 shows the response to HAI. Fifteen patients (83%) in the responding group showed good outcomes (CR, PR), while 3 (17%) in the non-responding group showed bad outcomes (NC, PD). There were no significant differences between the two groups in regard to pathology and diameter of liver metastasis. The duration of response in the responding group was 11.4 months. However, the duration of response in the responding group, except for one case who has survived for over 5 years, was 7.6 months. MST was 20.0 months in the responding group and 15.3 in non-responding group.

**Table 2:** response of HAI

	responding group CR, PR	non-responding group NC, PD	*p*-value
n	15	3	
Pathology			
differentiated	10	3	0.2393
undifferentiated	5	0	
Diameter of tumor (cm)	6.1 ± 1.9*	4.3 ± 3.2*	0.2099
Other metastasis	5	1	
Duration of response in all patients (mon)	11.4		
Duration of response (mon)	7.6		
(except one case who survive over 5 years)			
Median survival (mon)	20.0	15.3	

*: AVG ± SD

Multivariate analysis indicated that HAI therapy was an independent prognostic factor that influenced survival (p = 0.0120). However, except for the one case who survived for over 5 years, it was not an independent prognostic factor influencing survival (p = 0.0911). Other chemotherapies did not significantly contribute to prognosis (p = 0.9460).

**Figure 1:**
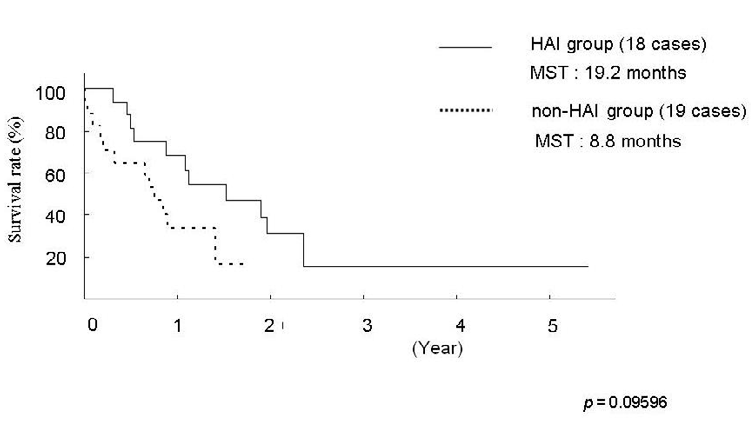
Survival curves of patients with synchronous and multiple liver metastases from gastric cancer.

Figure 1 shows a Kaplan-Meier survival curve. MST in the HAI group was 19.2 months and MST in the non-HAI group was 8.8 months; a more than two-fold difference that was, however, not significant (p = 0.09596). One patient in the HAI group survived for more than 5 years.

**Figure 2:**
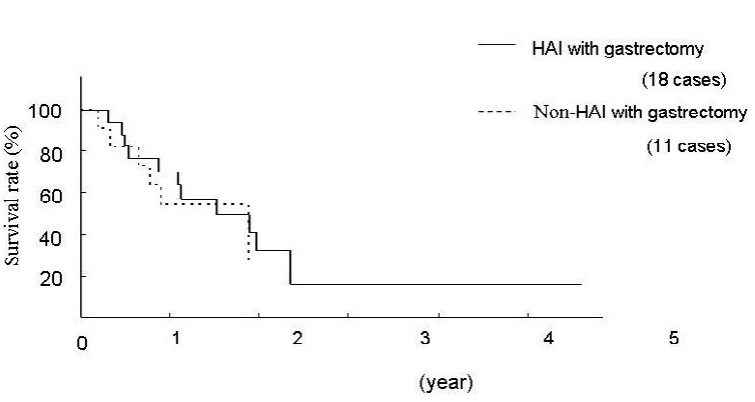
Survival curves of patients with multiple liver metastases from gastric cancer who underwent gastrectomy.

Figure 2 shows a Kaplan-Meier survival curve taking into account gastrectomy. Although all patients in the HAI group underwent gastrectomy, 8 patients (42%) in the non-HAI group did not undergo gastrectomy. Therefore, we examined the survival rate of the patients who had undergone gastrectomy. The survival curves from each group were found to correspond; therefore, the overall reduced survival rate in the non-HAI group shown in Figure 1 was considered to due to the patients without gastrectomy.

## Discussion

The prognosis for advanced gastric cancer has remained poor despite recent developments in chemotherapy. Although anti-cancer drugs are mainly administered intravenously, HAI is also performed [17-21]. HAI is not a systemic treatment but is an effective treatment for local organ providing a higher concentration to the tumor tissue and a lower one in the rest of the body. Therefore, HAI is expected to produce decreased side effects. The recurring type of advanced gastric cancer is complex and many cases of advanced gastric cancer have relapsed because of the combination of peritoneal dissemination, liver metastasis and/or lymph node metastasis. Local control, especially liver control, may be the most important strategy to extend survival. There are various HAI chemotherapy regimens. Several use a high dose of 5-FU and/or a combination of two or more anticancer drugs. In this study, the amount of 5-FU used was not so much, which enabled HAI to be continued for a long period. Although we did attempt to perform HAI chemotherapy with a high dose of 5-FU, we found we could not continue chemotherapy because of strictures and/or degeneration of the hepatic artery. Therefore, we selected a lower dose (see Methods). In this study, there were no severe side effects and the average period of HAI was 14.7 months. The MST of the HAI group was 19.2 months, thus HAI was used for a long period without side effects being demonstrated.

Although the overall response (CR + PR) rate was 83%, survival rate not extended. This response rate may be associated with an improvement of QOL; however, we did not evaluate the effect of HAI on QOL in this study so investigation is needed to evaluate the relationship. The prognosis of cases that underwent gastrectomy was good; that is, cases that could undergo gastrectomy achieved a good prognosis. The magnitude of the improvement in survival did not depend on HAI chemotherapy, the most important factor was the state of the patient.

## Conclusion

HAI is an effective treatment to control liver metastasis on its own. Local benefits to the liver were obtained by performing HAI for a long period of time, and the amount of 5-FU of 500 mg was an appropriate quantity. HAI was not associated with any improvement in the survival rate, which was conditional on the possibility of gastrectomy.

## Competing interests

The author(s) declare that they have no competing interests.

## Authors' contributions

**HO **– Design, Acquisition, analysis and interpretation of data, drafting manuscript, critical revision

**SO **– Data analysis

**TY **– Acquisition of data

**YM **– Acquisition of data

**YH **– Data analysis

**YN **– Data analysis, critical revision

**HK **– Critical revision

All authors read and approved the final manuscript
